# Role of the CCL2‐CCR2 signalling axis in cancer: Mechanisms and therapeutic targeting

**DOI:** 10.1111/cpr.13115

**Published:** 2021-08-31

**Authors:** Maosen Xu, Yang Wang, Ruolan Xia, Yuquan Wei, Xiawei Wei

**Affiliations:** ^1^ Laboratory of Aging Research and Cancer Drug Target State Key Laboratory of Biotherapy National Clinical Research Center for Geriatrics West China Hospital Sichuan University Chengdu China

## Abstract

The chemokine ligand CCL2 and its receptor CCR2 are implicated in the initiation and progression of various cancers. CCL2 can activate tumour cell growth and proliferation through a variety of mechanisms. By interacting with CCR2, CCL2 promotes cancer cell migration and recruits immunosuppressive cells to the tumour microenvironment, favouring cancer development. Over the last several decades, a series of studies have been conducted to explore the CCL2‐CCR2 signalling axis function in malignancies. Therapeutic strategies targeting the CCL2‐ CCR2 axis have also shown promising effects, enriching our approaches for fighting against cancer. In this review, we summarize the role of the CCL2‐CCR2 signalling axis in tumorigenesis and highlight recent studies on CCL2‐CCR2 targeted therapy, focusing on preclinical studies and clinical trials.

## Graphical abstract

The chemokine ligand CCL2 and its receptor CCR2 are implicated in the initiation and progression of various cancers. The CCL2‐CCR2 signalling axis plays a critical role in the promotion of pathological angiogenesis, the survival and invasion of tumour cells, and the recruitment of immune inhibitory cells. Therefore, CCL2 and CCR2 enable us to explore the sophisticated mechanisms underlying cancer development and provide potential options for treating malignant tumours.

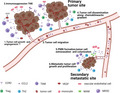

## 1 INTRODUCTION

Chemokines are small‐molecule proteins that exert their functions by binding to G protein‐coupled chemokine receptors (GPCRs) expressed on the cell surface.^1^, ^2^, ^3^ Initially, chemokines were described as mediators that induced immune cell infiltration and migration to specific inflammatory response sites. Subsequent studies revealed that chemokines participated in different biological processes, especially in the development of malignant tumours.^4^, ^5^ Chemokines can interact with tumour cells and the tumour microenvironment, promoting tumour occurrence and progression.^6^ Therapeutic methods targeting chemokines and their receptors have been developed from bench to bedside and have shown promising prospects.

To date, over 50 chemokines and 19 different chemokine receptors have been identified in human beings.^7^ According to the number and spacing of the conserved cysteine residues in the N‐terminus, chemokines are categorized into four main subfamilies: CXC, CC, C and CX3C.^3^, ^8^ Most chemokines belong to the CC and CXC subfamilies. In the CC‐chemokine sub‐family, a total of five monocyte chemoattractant proteins (MCP) have been identified: CCL2 (MCP‐1), CCL8 (MCP‐2), CCL7 (MCP‐3), CCL13 (MCP‐4) and CCL12 (MCP‐5).^9^, ^10^ Among them, CCL2 has similar sequence homology with other family members. For example, CCL8 and CCL7 have 62% and 71% amino acid identity with CCL2, respectively.^11^ After binding to their corresponding ligands, chemokine receptors undergo conformational changes that make G proteins bind to intracellular loop epitopes and the carboxy‐terminal tail of the receptors. Chemokine receptor activation induces a series of intracellular signals, resulting in cell motility and exerting diverse biological effects in the related target cells.^12^, ^13^

CCL2, also known as monocyte chemoattractant protein‐1 (MCP‐1), was initially isolated and purified from the culture supernatants of peripheral blood mononuclear cells and tumour cell lines in 1989.^14^, ^15^, ^16^ CCL2 was the first discovered and well investigated CC chemokine, preferentially binding to its receptor CCR2.^11^ Previous studies indicated that the CCL2‐CCR2 signalling axis played a role in the promotion of pathological angiogenesis, the survival and invasion of tumour cells, and the recruitment of immune inhibitory cells.^17^, ^18^, ^19^ Consequently, CCL2 and CCR2 enable us to explore the sophisticated mechanisms underlying cancer development and provide potential options for treating malignant tumours. In the present review, we introduce the mechanisms of the CCL2‐CCR2 axis in the process of tumorigenesis. We also focus on the research progress on the CCL2‐CCR2 axis in both preclinical studies and clinical trials.

## 2 BIOLOGICAL CHARACTERISTICS OF THE CCL2‐CCR2 SIGNALLING AXIS

CCL2 is a 13 kDa protein composed of 76 amino acids, and its coding gene is mapped at human chromosome 17 (chr. 17, q11.2).^11^, ^20^ A wide range of cells can produce CCL2, including tumour cells, endothelial cells, fibroblasts, epithelial cells, smooth muscle cells and myeloid cells.^21^ Moreover, CCL2 is able to regulate the infiltration and migration of various cells such as monocytes, memory T lymphocytes and natural killer (NK) cells, playing critical roles in the immune response.^22^

CCL2 mainly binds to the receptor CCR2. Structurally, the N‐terminal tail at the end of CCL2 is a significant determinant of CCR2 binding affinity and efficacy.^23^ Functionally, the binding of CCL2 and its cognate receptor CCR2 is essential for initiating signal transduction pathways and stimulating cell migration. In addition to CCR2, CCL2 can bind to other receptors. For example, the binding of CCL2 and CCR4 is involved in mammary gland development and promotes breast cancer progression.^24^ Moreover, CCL2 can bind to atypical receptors (ACKR1 and ACKR2). ACKRs and typical chemokine receptors differ structurally in the substituted DRYLAIV amino acid motif in the second intracellular loop of the ACKR. These atypical receptors cannot generate signals through G proteins and lack chemotactic activity. Therefore, they are often referred to as ‘scavenger receptors’.^25^, ^26^ Despite this, multiple research results have shown that CCR2 was the primary receptor of CCL2.^27^

**FIGURE 1:**
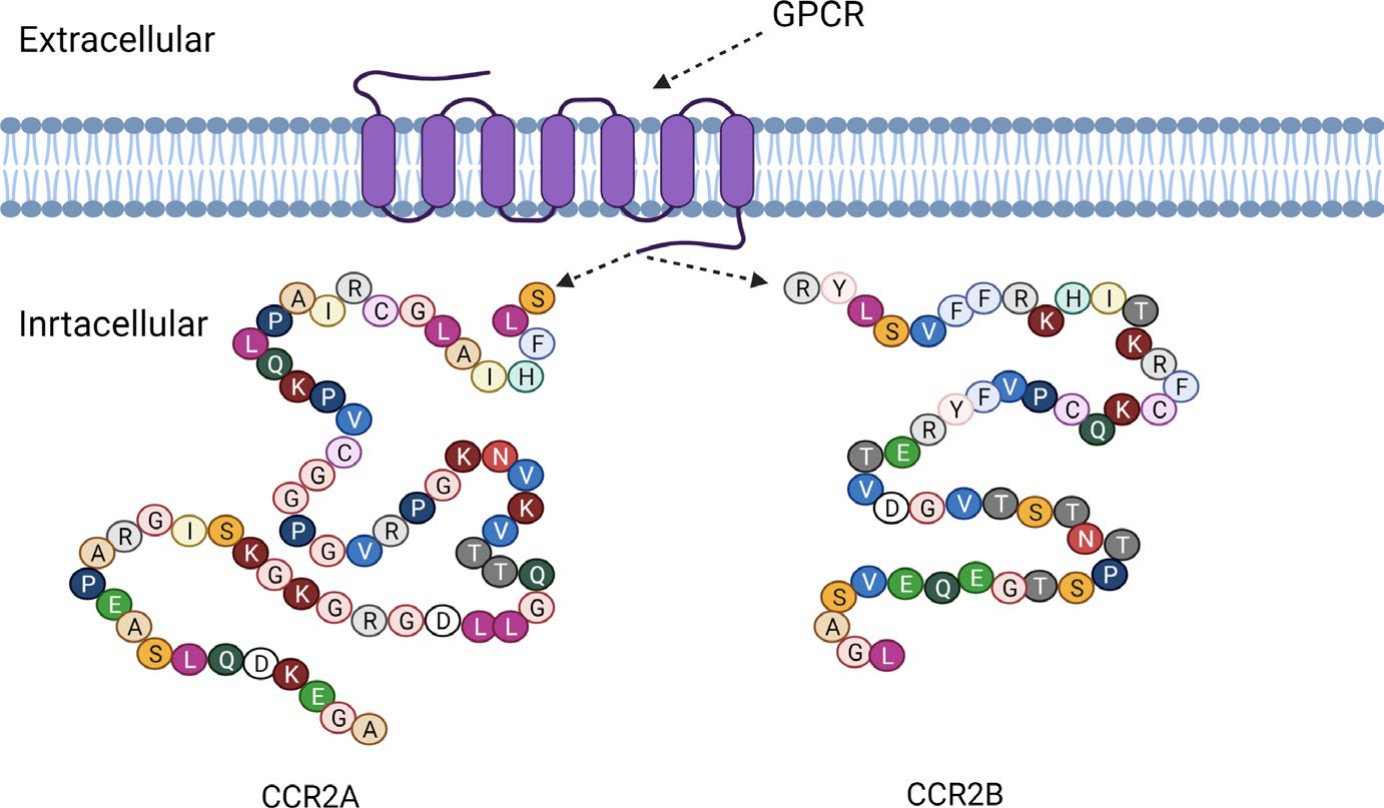
The structure of human CCR2A and CCR2B molecules. CCR2 belongs to the family of G protein‐coupled receptors (GPCRs) and exists as two splice variants that are CCR2A and CCR2B. CCR2A and CCR2B have been found to differ only in their terminal carboxyl tails

CCR2 belongs to the GPCRs and contains an N‐terminal extracellular domain and seven conserved transmembrane domains. Based on the difference in terminal carboxyl tails, CCR2 is divided into CCR2A and CCR2B (Figure 1), and they may function through different signalling pathways.^28^, ^29^ CCR2B is the predominant isoform of the CCR2 surface receptors and can be trafficked well to the cell surface, but CCR2A is detected predominantly in the cytoplasm.^30^ CCR2 functions by binding to different ligands, including CCL2, CCL7, CCL8, CCL12, CCL13 and CCL16, leading to the redundancy and promiscuity of the chemokine family. Nevertheless, it is this redundancy that has evolutionary significance for maintaining chemokine system activity and stability.^31^ Meanwhile, among the different ligands known to bind to CCR2, CCL2 has significantly higher activity than the other ligands.^32^

As the mainly functional receptor of CCL2, CCR2 is widely expressed by different types of cells, including dendritic cells (DCs),^33^ endothelial cells,^34^ monocytes^28^ and various cancer cells. Moreover, we also discover that CCR2 is expressed at low levels in neutrophils and lymphocytes.^35^ CCR2 upregulation is closely related to the recurrence and metastasis of advanced cancers. Once CCR2 is triggered by its ligand CCL2, various G protein‐mediated signalling cascades within the cell will be activated, such as the phosphatidylinositol 3‐kinase (PI3K)/AKT, mitogen‐activated protein kinase (MAPK)/p38 and Janus kinase (JAK)/STAT3.^36^, ^37^, ^38^ The activation of these signalling pathways plays a vital role in anti‐apoptosis, angiogenesis, and cell migration, leading to oncogenic advancement. The CCL2‐CCR2 axis is also implicated in the progression of numerous human diseases, such as inflammatory pain,^39^ atherosclerosis,^40^ neuroinflammatory diseases,^41^ rheumatoid arthritis^42^ and diabetic nephropathy.^43^ Therefore, the CCL2‐CCR2 axis is considered an appealing target for the treatment of these diseases.

## 3 ROLE OF THE CCL2‐CCR2 SIGNALLING AXIS IN CANCER PATHOGENESIS

It has been revealed that the CCL2‐CCR2 signalling axis participated in the occurrence and progression of a wide range of malignancies, such as breast cancer,^44^ prostate cancer,^45^ lung cancer,^46^ hepatocellular cancer,^47^ pancreatic cancer,^48^ nasopharyngeal carcinoma^49^ and renal cancer.^50^ Hence, numerous clinical studies have identified this axis as a predictor of early diagnosis and poor prognosis, which is shown in Table 1.

**TABLE 1:** Role of CCL2‐CCR2 axis as a prognostic factor in various cancers

Cancer types	Functions of the CCL2‐CCR2 axis
Prostate cancer	High serum CCL2 level is associated with poor overall survival and can be regarded as a diagnostic marker.^178^
Breast cancer	CCL2 expression is an independent risk factor for disease‐free survival (DFS) and correlated with poor prognosis.^105^
Lung adenocarcinoma	High expression of CCL2 is correlated with unfavourable overall survival (OS) and poor disease‐free survival (DFS).^175^
Gastric cancer	CCL2 can be a predictive biomarker for patients prognosis and tumour aggressiveness.^179^, ^180^
Colorectal cancer	High expression of CCL2 is associated with poor overall survival (OS) in colorectal cancer patients.^181^
Pancreatic cancer	High level of serum CCL2 is strongly associated with poor survival and prognosis.^182^, ^183^
Nasopharyngeal carcinoma	Overexpression of CCL2/CCR2 are significantly associated with poor overall survival (OS) in NPC patient.^184^, ^185^
Cervical cancer	CCL2 expression is a marker of cervical cancer, and enhanced expression of CCR2 may correlate with poor overall survival.^186^
Ovarian cancer	The overexpression of CCR2 shortens overall and progression‐free survival in patients with ovarian cancer.^187^
Liver cancer	CCL2 is an independent prognostic factor for patients and appears to be a promising complementary biomarker for HCC diagnosis.^188^, ^189^
Kidney cancer	The CCL2/CCR2 axis can be regarded as an independent prognostic factor for non‐metastatic clear‐cell renal cell carcinoma patients after surgical treatment.^50^, ^190^ Furthermore, high CCL2 expression is related to worse clinical stage and poor overall survival (OS) in patients with clear‐cell renal cell carcinoma.^191^
Bladder cancer	CCL2 expression is associated with poor overall survival (OS).^192^
Oesophageal cancer	The expression of CCL2 is an independent predictor of overall survival (OS) and predicts poor prognosis.^102^
Osteosarcoma	CCL2 expression is correlated with tumour prognosis in patients with osteosarcoma.^193^
Thyroid carcinoma	The expression of CCL2 is an independent predictive factor for recurrence of papillary thyroid carcinoma.^194^
Salivary adenoid cystic carcinoma	The overexpression of CCL2 is associated with the clinical progression and poor prognosis of SACC.^104^
Glioma	CCL2 overexpression is correlated with the reduced overall survival of patients.^61^

**FIGURE 2:**
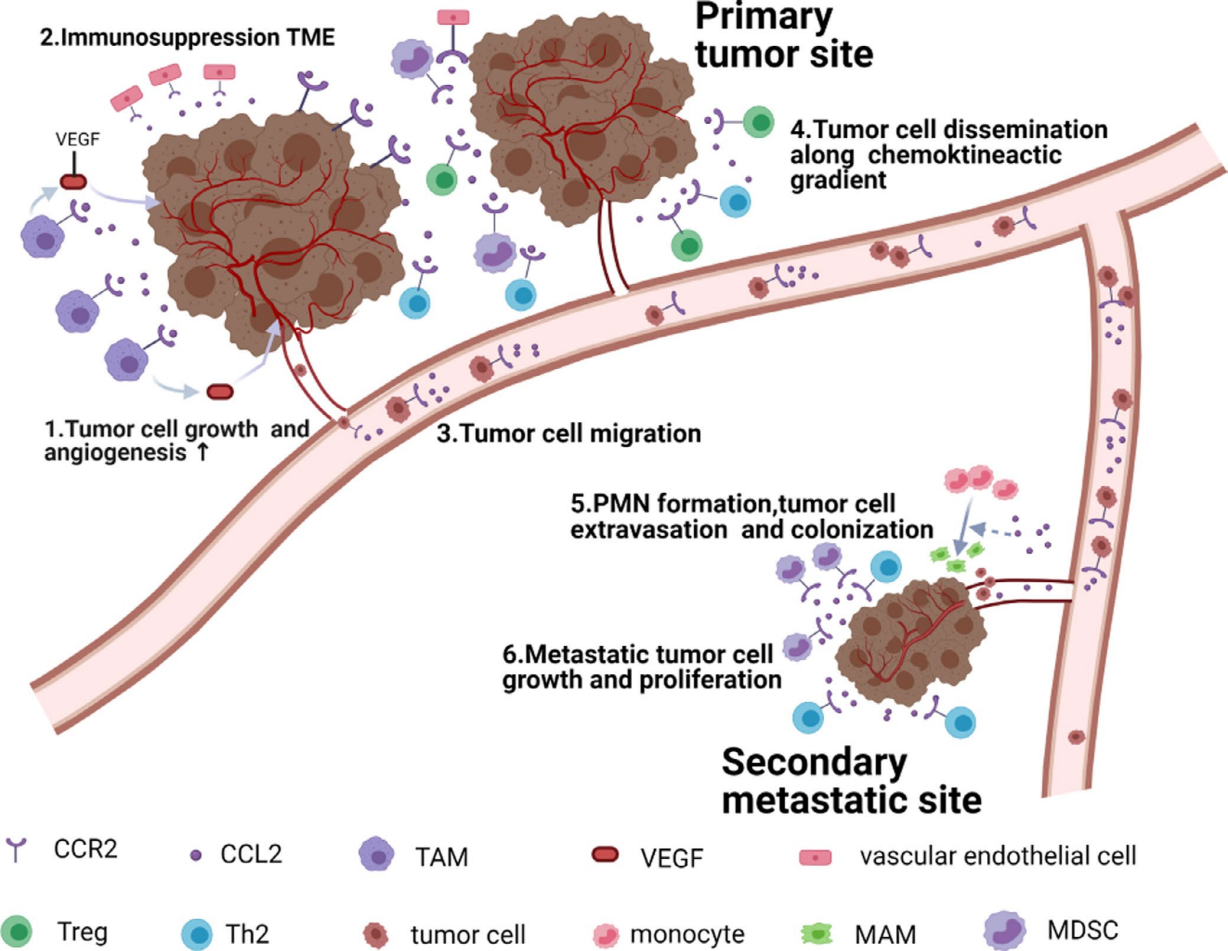
The role of the CCL2‐CCR2 axis in tumour development. CCL2 is mainly produced by tumour cells and surrounding stromal cells. By interacting with CCR2, CCL2 sustains tumour cell growth and proliferation. CCL2 is capable of recruiting CCR2^+^ TAMs, MDSCs and Th2 cells to create an immunosuppression microenvironment that contributes to tumour progression. Besides, TAM‐secreted vascular endothelial growth factor (VEGF) promotes tumour angiogenesis through the interaction with CCR2^+^ vascular endothelial cells. In the secondary sites, CCL2 facilitates the differentiation of monocytes into MAMs and the formation of pre‐metastatic niche, accelerating metastatic tumour cell colonization and growth

Tumorigenesis is a dynamic and complicated process that consists of three stages including initiation, progression and metastasis.^51^ The CCL2‐CCR2 signalling axis participates in different stages of tumorigenesis. The axis sustains tumour cell growth and proliferation at the primary tumour site. When malignant cells break away from their original locations to metastasize, the CCL2‐CCR2 axis can stimulate cancer cells to invade surrounding tissues, entry to the circulatory system and spread along a specific chemotactic gradient to metastatic sites.^17^ After reaching a specific secondary organ and/or tissue, surviving circulating tumour cells can colonize successfully and continue to grow by interacting with the tumour microenvironment. A diagram of the mechanism of CCL2‐CCR2‐mediated tumour pathogenesis is shown in Figure 2.

### 3.1 Tumour growth and proliferation

Primary tumour growth is a multi‐step and complicated process involving various growth factors and signalling pathways regulation. Previous studies have shown that both stromal‐ and tumour‐derived CCL2 could directly stimulate tumour cell growth and proliferation.^36^, ^52^ Lu et al^53^ found that CCL2 might accelerate tumour growth by acting as an autocrine or paracrine growth factor. Moreover, CCL2 promoted cancer cell to achieve self‐renew and reproduce by activating a series of signal transduction pathways downstream of GPCRs.

**FIGURE 3:**
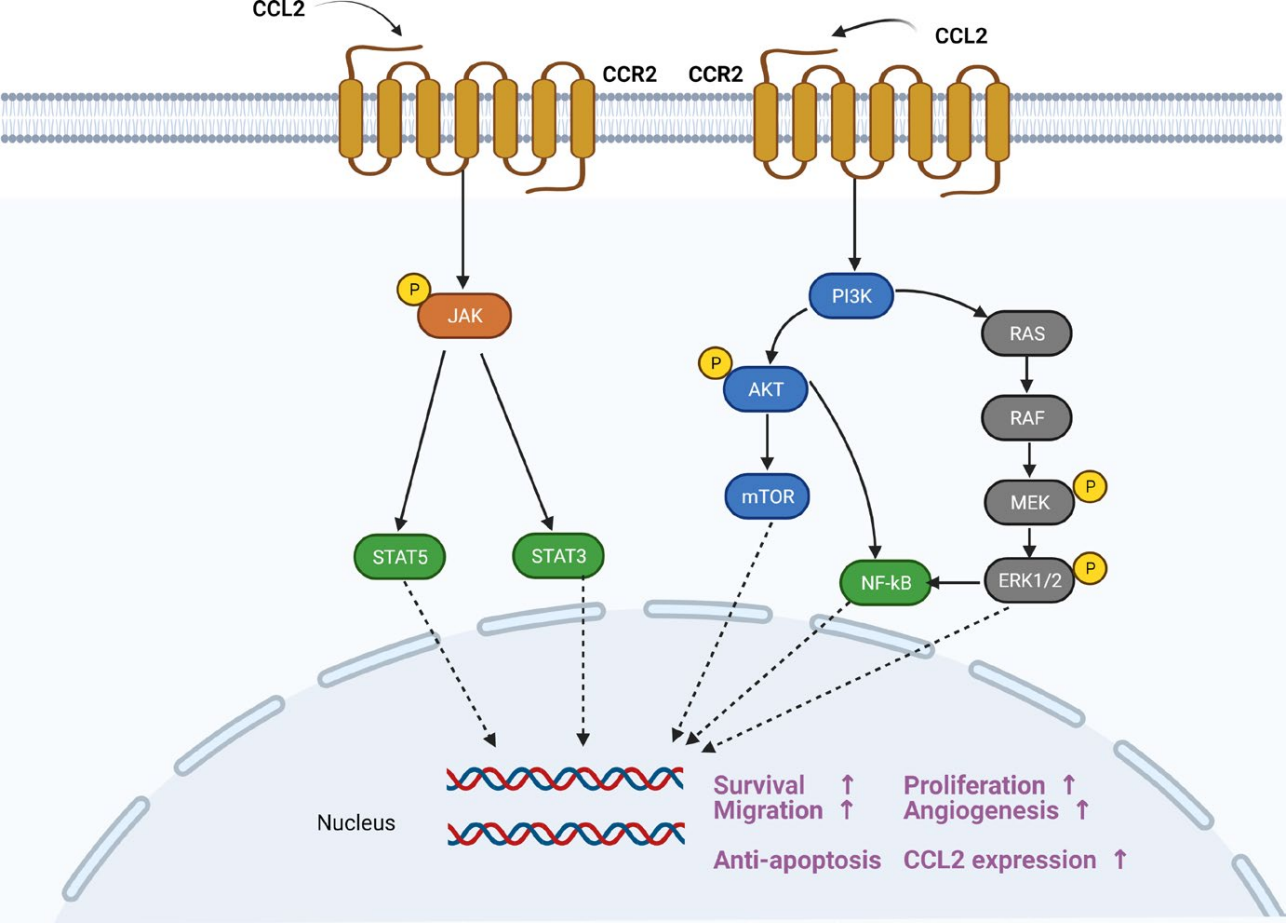
The CCL2‐CCR2 axis and transduction pathway. CCR2 is a typical G‐protein‐coupled receptor. When CCR2 binds to its ligand CCL2, a series of downstream signals are activated, such as JAK/STAT, p38MAPK and PI3K/AKT. Then, multiple transcription factors and genes are mobilized, leading to tumour cell growth, proliferation, migration and the increased CCL2 expression

Different signalling pathways activated by the CCL2‐CCR2 axis are involved in tumorigenesis (Figure 3). It was reported that CCL2 could protect prostate cancer PC‐3 cells from autophagic death and prolong the survival of tumour cells in serum‐free conditions via activating PI3K/AKT signalling.^54^, ^55^ What is more, activated PI3K/AKT signalling remarkably promoted the expression of Survivin, a small protein belonged to the inhibitor of apoptosis protein family, suppressing both autophagy and apoptosis of cancer cell. Moreover, CCL2 hindered the activation of docetaxel‐induced apoptosis‐associated protein caspase‐3 to support tumour cell growth. This process was also correlated with the PI3K/AKT pathway activation, and CCL2 overexpression contributed to the activation of AKT by promoting its phosphorylation at Ser473.^56^ In addition, CCL2 regulated cancer cell viability, motility and survival by the activation of mitogen‐activated protein kinase (MAPK).^57^ CCL2 derived from cancer‐associated mesothelial cells could elevate ovarian cancer malignant potential, which was dependent on p38‐MAPK signalling.^38^ In vitro experiments, researchers demonstrated that recombinant hCCL2 dramatically promoted cancer cell proliferation by activating the MAPK/ERK pathway and enhanced the MEK and ERK1/2 phosphorylation levels.^58^

One of the major issues with cancer eradication is the acquisition of resistance to both chemotherapies and radiotherapies in due course treatment. CCL2 can decrease drug‐induced cytotoxicity to support tumour cell growth by inhibiting proapoptotic autophagy and is regarded as an intervention target for chemotherapy resistance.^59^ Similarly, CCL2 also contributes to the chemoresistance of lung cancer cells to docetaxel, which may be attributed to cell stress responses.^56^ Furthermore, ablative radiotherapy increased the production of tumour‐derived CCL2 in a pancreatic ductal adenocarcinoma model, and the increased CCL2 promoted the recruitment of Ly6C^+^CCR2^+^ monocytes in tumour sites, thereby accelerating tumour development.^60^

CCL2 can also recruit various immune cells (such as myeloid‐derived suppressor cells, MDSCs) to form an immunosuppressive microenvironment, which allows tumour cell to evade the body's immune surveillance and supports tumour cell proliferation.^61^, ^62^ Overall, the tumorigenic processes mediated by the CCL2‐CCR2 axis involve a series of cellular or molecular signalling, the acquisition of chemoresistance and radioresistance, and the recruitment of multiple immunosuppressive cells. Therefore, more attention should be paid to comprehensively investigate the underlying action mechanisms of the CCL2‐CCR2 axis in tumour proliferation in the future.

### 3.2 Tumour angiogenesis

Tumour angiogenesis is a critical process in tumour progression and consists of tightly regulated events that include basement membrane degradation, endothelial cell migration and proliferation, and capillary tube formation. Angiogenesis also involves various stimulatory factors such as vascular endothelial growth factor (VEGF).^63^ In cancer, tumour cells acquire invasive behaviours and induce robust angiogenesis.^64^ Based on the more profound understanding of the CCL2‐CCR2 axis, scientists found that this axis could act as a mediator of angiogenesis and lead to extensive neovascularization. The CCL2‐CCR2 axis can mediate pathological angiogenesis regulation both directly and indirectly.

CCL2 could bind to CCR2 expressed on vascular endothelial cells and directly stimulated angiogenesis in the absence of inflammatory infiltration. Treatment with a CCL2 neutralizing antibody showed obvious inhibition of the CCL2‐modulated angiogenic effect and reduced the metastatic lesions.^65^ CCL2 also stimulates angiogenesis through the activation of transcription factors. Recently, Stamatovic et al^66^ demonstrated that the transcription factor Ets‐1 played a crucial role in CCL2‐mediated angiogenesis. CCL2 probably exerted its angiogenic activity by upregulating Est‐1 target molecules such as β_3_ integrin. Furthermore, CCL2‐mediated Est‐1 activation was associated with the ERK1/2 cascade, and the inhibition of ERK1/2 with PD98059 obviously decreased Ets‐1 phosphorylation and then inhibited angiogenesis.

On the other hand, CCL2 induced angiogenesis through indirect ways that were correlated with the recruitment of inflammatory cells.^67^, ^68^ Tumour‐associated macrophages (TAMs) recruited by CCL2 upregulated the expression of angiogenic factors such as VEGF. In a rat glioblastoma model, TAMs recruitment was blocked after the administration of CCL2 inhibitor, thus reducing angiogenesis and inhibiting tumour growth.^69^ High expression levels of matrix metalloproteinase‐9 (MMP9) also contributed to tumour vascularization. After CCL2 administration, intratumoral MMP9 expression was increased significantly and the survival time was declined dramatically in tumour‐bearing mice.^70^

In brief, CCL2 promotes tumour angiogenesis by recruiting CCR2^+^ vascular endothelial cells and inflammatory cells and by stimulating the expression of angiogenic factors. Based on the finding that CCL2 inhibition limits tumour angiogenesis,^71^ we presume that CCL2 may be a potential anti‐angiogenesis target in cancer therapy.

### 3.3 Tumour metastasis

Tumour metastasis is an integrated multi‐step process by which cancer cells disperse from the original tumour sites through the blood or lymphatic system to the surrounding tissues or distant organs, and remains the leading cause of death among cancer patients.^72^ Metastasis involves a series of events regarding epithelial‐mesenchymal transition (EMT), tumour cell invasion of the basement membrane and entry into the bloodstream to form circulating tumour cells (CTC), formation of pre‐metastatic niches, homing of cancer cell and tumour growth at the metastatic site.^73^, ^74^ The CCL2‐CCR2 signalling axis contributes to cancer cell abscission, invasion and migration, playing a pivotal role in tumour metastasis.

#### 3.3.1 The CCL2‐CCR2 axis induces epithelial‐mesenchymal transition

At the initial stage of metastasis, tumour cells acquire an invasive phenotype that can break down the extracellular matrix (ECM) to facilitate metastasis.^17^ Then, epithelial‐mesenchymal transition (EMT) reduces the adhesion between cells and facilitates cell crossing of the basement membrane.^75^ Numerous studies have shown that the EMT programme was involved in modulating the metastasis and spread of diverse tumour cells. IL‐6, a cytokine, was reported to support tumour cell invasion by inducing EMT effectively. Besides, the addition of CCL2 dramatically enhanced this EMT‐like morphological change compared with IL‐6 alone, which was associated with the phosphorylation of STAT3.^76^ Pharmacological interruption of the CCL2‐CCR2‐STAT3 signal markedly suppressed EMT and decreased tumour cell migration.^37^ Furthermore, CCL2 induced EMT through the upregulation of transcription factor Snail and the activation of the Hedgehog pathway.^47^, ^77^

Matrix metalloproteinases (MMPs) are a multigene family of metal‐dependent endopeptidases that play important roles in EMT and contribute to tumour metastasis.^78^ The CCL2‐CCR2 signalling axis upregulates MMP2 and MMP9 expression to promote cancer cell infiltration and migration. In a human chondrosarcoma model, MMP9 expression was increased in CCL2‐treated cells. However, after the transfection of MMP9‐small interfering RNA(siRNA), MMP9 expression was inhibited and the CCL2‐mediated tumour cell migration was also reduced.^79^ NF‐κB was a highly conserved transcription factor and was also responsible for enhancing CCL2‐mediated MMP‐9 expression and cell motility.^80^ Besides, MMP2 was discovered to engage in CCR2‐mediated EMT, whereas CCR2‐siRNA transfection could reduce MMP2 activity, indicating the specific binding between MMP2 and CCR2.^81^

#### 3.3.2 The CCL2‐CCR2 axis induces tumour cell extravasation

When tumour cells enter the lymphatic system or bloodstream, they become circulating tumour cells (CTCs) and disseminate along chemotactic gradients. In the new sites, tumour cells exude and continue to colonize. This step is associated with the interactions between the host factors in the target organs and the spreading tumour cells. In tumour cell extravasation, scientists found a population of cells derived from a subset of inflammatory monocyte precursors, known as metastasis‐associated macrophages (MAMs).^82^, ^83^ A study conducted by Kitamura et al^84^ indicated that CCL2‐CCR2 axis activation was able to promote CCL3 secretion by recruited MAMs. Meanwhile, the secretion of CCL3 led to enhanced interaction between MAMs and cancer cells and prolonged MAMs retention in tumour sites, which accelerated the extravasation of malignant cells. In addition, CCR2 deficiency prevented tumour cell extravasation. Researchers also noticed that the phosphorylation of JAK2 was increased in lung homogenates from MC‐38‐injected mice but no or minor JAK2 phosphorylation was detected in CCR2^−/−^ mice. Similarly, STAT5 and p38MAPK could not be activated in CCR2^−/−^ tumour tissues after tumour cell injection, indicating that CCL2‐induced tumour cell migration had a close relationship with JAK2‐STAT5 and p38MAPK pathway activation.^85^

#### 3.3.3 The CCL2‐CCR2 axis induces the homing of tumour cell

After malignant cell exudation from the circulatory system, their growth in secondary sites is also critical for cancer metastasis. Dating back to the 1980s, the English surgeon *Stephen Paget* proposed the ‘seed and soil’ hypothesis, which suggested that the spread of tumour cells was controlled by the interaction between the cancer cells (seed) and the host organ (soil).^86^, ^87^ Subsequently, in 2005, Kaplan and colleagues discovered that bone marrow‐derived hematopoietic progenitor cells clustered to tumour‐specific pre‐metastatic sites and formed cellular clusters before tumour cells arrived, which indicated the existence of ‘pre‐metastatic niche (PMN)’.^88^

The PMN is considered as fertile soil for the survival and growth of metastatic tumour cell and represents an aberrant, tumour growth‐favouring microenvironment.^86^, ^89^ It has been reported that CCL2 contributed to the establishment of the PMN and the homing of tumour cells. CCL2 secreted by stromal cells or tumour cells could recruit inflammatory monocytes to stimulate PMN formation and create a suitable environment for cancer cell colonization.^90^ Intriguingly, in a hypoxic environment, CCL2 induced by tumour cells may inhibit the maturation of natural killer cells in PMN and reduce the ability of NK cells to eliminate incoming CTCs, accelerating the homing of cancer cells.^91^

In both the early and late stages of metastasis, the CCL2‐CCR2 signalling axis also recruits diverse immune cells to form an immunosuppressive TME that allows tumour cells to evade the body's immune surveillance, which will be discussed in the next section. Altogether, CCL2 and its receptor CCR2 are upregulated in tumours and engaged in tumour metastasis through the induction of EMT, the promotion of tumour cell extravasation and the establishment of a PMN.

## 4 ROLE OF THE CCL2‐CCR2 AXIS IN THE TUMOUR MICROENVIRONMENT

**FIGURE 4:**
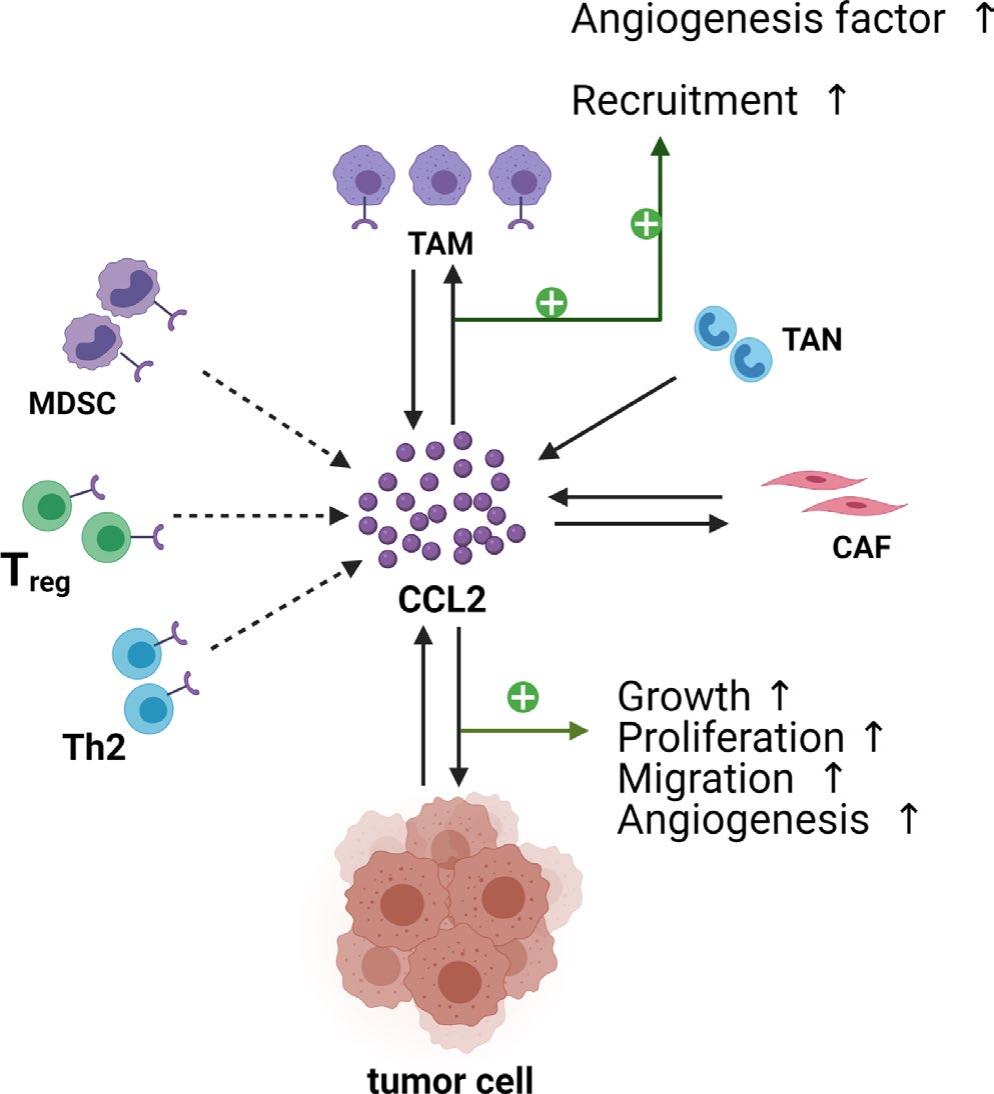
CCL2 and the tumour microenvironment. In the tumour microenvironment, cancer cells interact with various cells to promote the occurrence and development of tumours. CCL2 can recruit various immunosuppressive cells to the TME and weaken the anti‐tumor immune response. In addition to tumour cells, other cells are able to secrete CCL2, such as TAMs, TANs and CAFs

In the past few decades, cancer researches mainly focused on malignant cells and related gene regulation. More recently, the concept of the tumour microenvironment (TME) is becoming widely accepted. The dynamic interplay between the tumour and its microenvironment has a significant influence on carcinogenesis. Other than tumour cells, the TME is composed of a great variety of nonmalignant cells such as fibroblasts, mesenchymal stem cells, haemolymph endothelial cells and numerous infiltrating leukocytes.^92^ Some of these cells can upregulate CCL2 expression and participate in tumour progression. Herein, we will discuss the relationship between the CCL2‐CCR2 axis and these nonmalignant cells in the TME, with an emphasis on immune cells and fibroblasts (Figure 4).

### 4.1 Macrophages

As one of the leucocytes in the innate immune system, macrophages exist in nearly all organs and tissues. In the events of tissue injury, infection and cancer, monocytes are recruited to the related sites by chemotactic signals and further differentiate into macrophages to participate in the body's immune response.^93^ Macrophages can differentiate into two different types: classically activated (or inflammatory) macrophages (M1) and alternatively activated (or wound‐healing) macrophages (M2), a process termed ‘macrophage polarization’.^94^ Generally, M2 macrophage is characterized in vitro by the production of IL‐12^low^ IL‐23^low^ IL‐10^high^ TGF‐β^high^, whereas M1 macrophage is characterized by IL‐12^high^ IL‐23^high^ IL‐10^low^.^94^ Furthermore, a frequently used marker to identify M2 macrophages is Arginase‐1, whereas Arginase‐1 is also induced in M1 spectrum macrophages. Therefore, scientists usually favour an approach using combinations of markers (or a lack of marker expression) to identify macrophage subsets.^95^ For instance, the analysis of the transcriptional signature of murine M1 and M2 macrophages showed that CD38, G protein coupled receptor 18 (Gpr18) and Formyl peptide receptor 2 (Fpr2) are useful markers for M1, whereas Early growth response protein 2 (Egr2) and c‐Myc were M2‐exclusive.^96^ Functionally, M1 macrophages promote inflammation and exhibit anti‐tumour properties. In contrast to M1, M2 macrophages play an immunosuppressive role that is conducive to tissue repair and promotes tumour progression.^97^ Tumour‐associated macrophages (TAMs) are a type of macrophages aggregated in the microenvironment of tumour‐infiltrating tissues and are mainly differentiated from bone marrow‐derived mononuclear cells.^98^ TAMs are generally considered as a specialized phenotype of M2‐like macrophages as they have similar functions with M2 macrophages and facilitate tumour growth by inducing immune suppression.^99^, ^100^

The CCL2‐CCR2 axis promotes monocytes' recruitment from the peripheral blood to the tumour sites, where these monocytes further differentiate into TAMs.^99^, ^101^ TAMs accelerate tumour proliferation and metastasis by inducing immune escape. Yang et al^102^ found that in oesophageal cancer, tissues with high CCL2 expression showed a corresponding increase in the number of cells expressing CD68 (a common TAM marker). Besides, increased expression of PD‐L2 in TAMs supported immune escape through the PD‐1 pathway. In metastatic colorectal cancer, a large number of TAMs infiltrate the liver to facilitate metastatic cancer cell growth, which is driven by TAM‐mediated immunosuppression.^103^ Intriguingly, not only does the CCL2‐CCR2 axis promote tumour progression by recruiting and reprogramming TAMs,^104^ but TAMs also support cancer invasion and metastasis by secreting CCL2.^105^ Such crosstalk and mutual promotion provide a positive induction for TAMs in the TME and establish favourable conditions for tumour growth and metastasis.

### 4.2 Neutrophils

Neutrophils are derived from a common committed myeloid progenitor cell and are considered as effectors of the innate immune response. Neutrophils coordinate multiple immune processes including chronic inflammatory, autoimmunity and cancer.^106^ Meanwhile, neutrophils have been uncovered in the TME of various cancers. Tumour‐associated neutrophils (TANs), similar to macrophages, have anti‐ and pro‐tumour heterogeneities modulated by the TME.^107^, ^108^ The extensive TAN infiltration was observed to have a positive relationship with the advanced stage and poor prognosis of oral squamous cell carcinoma. Melatonin could delay cancer progression by inhibiting CCL2 release from TANs.^109^ Furthermore, CCL2‐positive TANs were widely distributed in the primary tumour sites and regional lymph nodes,^110^ suggesting that they might facilitate tumour progression through CCL2 expression.

### 4.3 Myeloid‐derived suppressor cells

Myeloid‐derived suppressor cells (MDSCs) were initially described in the 1980s and accumulated in the TME.^111^ MDSCs are a group of immature immunosuppressive cells derived from the bone marrow, and their notable function is to suppress T‐cell response.^112^, ^113^ Generally, MDSCs are divided into two types: polymorphonuclear MDSC (PMN‐MDSC), which are morphologically and phenotypically similar to neutrophils, and another is monocytic MDSC (M‐MDSC) that is similar to monocytes.^114^, ^115^ The CCL2‐CCR2 axis is required for MDSCs functional specialization. CCL2 promoted the progression of colorectal cancer and enhanced the polymorphonuclear MDSC population and function. Mechanismly, CCL2 regulated T‐cell‐suppressive activity mediated by PMN‐MDSC through the activation of STAT3 and the reactive oxygen species (ROS) production in PMN‐MDSC.^116^ Moreover, the CCL2‐CCR2 axis modulated the migration of M‐MDSC to tumour tissues and metastatic environment.^117^

Previous studies showed that the CCL2‐CCR2 axis also played a key role in the recruitment of MDSCs into the TME, which protected tumour cells from immune‐mediated killing.^118^ In a murine glioma model, one week after GL261 cells had been injected into mice, flow cytometric analysis of the tumour tissues showed that CCR2 was largely localized to MDSC populations. These CCR2^+^ MDSCs were recruited to the glioma microenvironment in response to CCL2, whereas MDSCs lacking CCR2 failed to maximally accumulate in the TME.^61^ Furthermore, MDSCs in the microenvironment prevented the influx of CD8^+^ T cell. Meanwhile, the CD8^+^T cell/MDSC ratio was increased within tumours derived from CCL2 deletion mice, and elevated levels of CD8^+^ T cells restored anti‐tumour immunity to inhibit tumour development.^119^

### 4.4 T cells

T cells are leukocytes that express T‐cell receptors (TCRs). T cells are divided into two types: CD8^+^ cytotoxic lymphocytes (CTL) that recognize peptides presented by MHC I and CD4^+^ helper (Th) cells that recognize peptides presented by MHC II.^120^ There are several subtypes of CD4^+^ T cell, among which Th1 and Th2 cells are most common and important. Th1‐derived cytokines induce pro‐inflammatory responses and exhibit anti‐tumour effects, whereas Th2‐secreted cytokines produce anti‐inflammatory effects and support tumour progression.^120^, ^121^

The secretion and expression of CCL2 is associated with Th2 polarization.^122^ CCR2 can be expressed in Th2 cells, and the CCL2‐CCR2 axis may facilitate Th2 effector cells' differentiation and maturation through the reduction of IL‐12 produced by antigen presenting cells and the increase of IL‐4 secreted by activated T cells.^123^ Moreover, CCL2 secreted from the TME could promote Th2 cell aggregation. In concert with this, researchers found that the ratio of Th1/Th2 was prominently decreased within tumour tissues of high CCL2 expression.^124^

In nearly all cancers, the number of regulatory T cell (Treg) is typically increased. Treg is a type of CD4^+^ T cell, and can foster tumour metastasis and progression by coordinating immunosuppressive effects.^125^ The majority of studies showed that the CCL2‐CCR2 axis was crucial for Treg recruitment and accumulation in the TME.^126^, ^127^ In a murine model of head and neck squamous cell carcinoma, the elevation of CCL2 after radiotherapy was involved in oncogenic transformation and further mediated the recruitment of CCR2^+^ Treg.^128^ Further, CCR2^+^ Treg represented high immunosuppressive activity and played a central role in suppressing anticancer responses. Although CCR2^+^ Treg accumulated in various cancers, it could be depleted by low‐dose cyclophosphamide, which allowed the definition of a novel target for improving chemotherapic efficacy.^129^

### 4.5 Fibroblasts

Cancer‐associated fibroblasts (CAFs) are one of the most abundant stromal components in the TME. Although their origin remains unclear, it is generally believed that CAFs might result from the activation of local tissue‐resident fibroblasts.^130^, ^131^ Shen et al^132^ revealed that miRNAs‐mediated FoxO3a/VEGF/CCL2 signalling played a prominent role in converting normal fibroblasts (NFs) into CAFs. The authors further indicated that CCL2 upregulation had a potential promoting effect on CAFs production. Intriguingly, CAFs also foster tumour metastasis by secreting CCL2. The overexpression of NF‐κB and STAT3 considerably stimulated the CCL2 secretion in cocultured CAFs.^133^, ^134^ Besides, in pancreatic cancer, tumour cells educate fibroblasts via the secretion of IL‐8 and CCL2, leading to the generation of CAFs and similar metastasis‐associated fibroblasts (MAFs).^135^

Collectively, the above results suggest that the CCL2‐CCR2 axis is upregulated during cancer progression and has a close relationship with cancer initiation, progression and metastasis. Blockade of the CCL2‐CCR2 axis is a potential therapeutic option for cancer treatment.

## 5 CCL2‐CCR2‐ASSOCIATED TARGETED THERAPY

The CCL2‐CCR2 signalling axis has a multifaceted involvement in a wide range of cancers, and accumulated experimental evidence is paving the way for future clinical research. Furthermore, blockade of the CCL2‐CCR2 axis has attracted extensive attention and represents promising anti‐tumour activity in both preclinical studies and clinical trials. Here, we mainly report the therapeutic strategies targeting the CCL2‐CCR2 axis.

### 5.1 Preclinical studies targeting CCL2‐CCR2 axis

#### 5.1.1 Inhibition of CCL2

A number of preclinical studies have used various CCL2 inhibitors or antibodies to delay tumour progression. Blockade of CCL2 achieves tumour suppression through diverse ways such as blocking CCL2‐mediated signalling pathways, inhibiting the immunosuppressive cell recruitment and increasing the number of tumour‐killing cells.

##### C1142

C1142 is a rat/mouse chimeric monoclonal antibody that can specifically neutralize CCL2. This antibody has yielded encouraging results in different cancer models as a single agent or combination with standard chemotherapies.^136^ In a glioma model, C1142 treatment significantly reduced the total numbers of CD11b^+^CD45^+^ TAMs and CD11b^+^Gr1^+^ MDSCs, inhibiting tumour proliferation and prolonging the survival of glioma‐bearing mice.^137^ C1142 also inhibited tumour cell migration by blocking the infiltration of TAMs into tumour sites.^138^ In addition, C1142 also enhanced hepatic NK‐cell cytotoxicity and increased IFNγ production, resulting in the inhibition of tumour progression.^139^ Administration of C1142 along with anticancer vaccine augmented CD8^+^ T‐cell numbers and reduced tumour volume, suggesting that combining CCL2 antibody with the vaccine could be a promising tactic for treating cancer.^140^

##### Bindarit

Bindarit is an anti‐inflammatory indazole derivative that can inhibit the synthesis of CCL2, with a potential inhibitory function in tumour development. Targeting CCL2 with bindarit induces tumour regression, which has been confirmed in some preclinical studies. Bindarit is able to impair the organization of endothelial cells in the formation of blood vessels to inhibit tumour angiogenesis.^141^ Moreover, bindarit impairs the TME by preventing TAM and MDSC infiltration, which limits tumour growth and delays tumour progression.^142^, ^143^ Further, bindarit inhibited tumour cell proliferation and migration in vitro through negative regulation of the NF‐κB and AKT signalling pathway.^142^

##### mNOX‐36

A CCL2 inhibitor, mNOX‐36 suppressed TAM recruitment in a rat glioblastoma multiforme model. Interestingly, the combination therapy of mNOX‐36 and bevacizumab reduced both the tumour size and blood volume.^69^ The safety and validity of mNOX‐36 require further evaluation in clinical trials.

##### Curcumin

The expression and activity of CCL2 can be inhibited by curcumin, an active polyphenolic compound of the powdered turmeric (*Curcuma longa*) root. In a mouse model of colon cancer, plasma CCL2 was reduced after curcumin feedings.^144^ Protein kinase C (PKC), an important intracellular signalling mediator, is known to upregulate CCL2 secretion and is associated with tumour cell invasion. Curcumin significantly blocks CCL2‐induced cell invasion and motility through the inhibition of PKC. Furthermore, Curcumin also reduces CCL2‐mediated MMP9 expression that contributes to tumour progression.^145^

##### Carlumab

Carlumab, also known as CNTO 888, is a human anti‐CCL2 antibody. Carlumab inhibits PC‐3 cell proliferation and migration in vitro. Investigators found that the combination of carlumab and docetaxel was more efficacious than docetaxel alone by inducing tumour regression.^146^ To date, using carlumab to treat cancer has been investigated in clinical trials, and we will discuss its efficacy in the related section.

#### 5.1.2 Inhibition of CCR2

CCR2 is overexpressed in immunosuppressive cells and tumour cells, but its relatively low expression in most normal tissues make this receptor a promising target in cancer.

##### RS 504393

RS 504393 is a selective CCR2 antagonist that delays tumour progression by inhibiting the infiltration of immunosuppressive cells into tumour sites. Researchers found that RS 504393 improved the survival time of mice with bladder cancer by blocking the recruitment of MDSCs.^147^ Following RS 504393 treatment, the number of TAMs and the tumour volume were significantly reduced in tumour‐bearing mice,^104^ suggesting that targeting CCR2 with RS504393 might be a potential therapeutic option for cancer.

##### RS 102895

RS 102895 is a CCR2 antagonist that blocks CCL2 signalling through the occupation of a binding site on the extracellular side of CCR2.^148^ RS 102895 treatment reduced prostate cancer PC‐3 cell invasion from 197 to 85 percent.^149^ Moreover, CCL2 regulated breast cancer cell growth and migratory ability, whereas RS 102895 could attenuate these regulatory effects partially due to the downregulation of MMP‐9 expression.^150^

##### CAS445479‐97‐0

CCL2 promotes lung cancer cells' growth, migration and invasion. CAS445479‐97‐0, a CCR2 antagonist, reversed the induction of A549 cell proliferation by CCL2 in a dose‐dependent manner. In addition, CAS445479‐97‐0 was able to inhibit A549 cell migratory capacity mediated by CCL2, which could be attributed to the downregulation of MMP‐9 expression.^151^ Therefore, targeting CCR2 with CAS445479‐97‐0 may be an attractive therapeutic strategy for patients with lung cancer.

##### GMME1

GMME1 is a novel fusion protein that functions as a CCR2‐specific tumoricidal agent. GMME1 is composed of granulocyte macrophage colony‐stimulating factor (GM‐CSF) in tandem with N‐terminal truncated monocyte chemotactic protein‐1 (MCP1 6‐76).^152^ Rafei et al^152^ developed it and confirmed its anti‐tumour activity. GMME1 specifically blocked CCR2‐associated STAT3 phosphorylation and induced tumour cell apoptosis, thereby inhibiting tumour proliferation.

##### Teijin Compound 1

Teijin Compound 1(TC1) is a specific CCR2 antagonist and is developed to block the binding of human CCL2 to CCR2b.^153^ In a trans‐endothelial tumour cell migration experiment, TC1 was observed to inhibit cancer cell migration. TC1 also reduced vascular permeability and prevented the generation of pulmonary metastases.^154^

##### BMS CCR2 22

BMS CCR2 22 is a potent and high affinity CCR2 antagonist. In a metastatic liver tumour model (MC38), BMS CCR2 22 treatment decreased the infiltration of TAMs into tumour sites effectively and increased the efficacy of FOLFOX chemotherapy, thereby improving the survival time of tumour‐bearing mice.^155^

##### 747

Recently, Yao et al^156^ reported a natural product from *Abies georgei*, named 747. 747 exhibited sensitivity and selectivity as a CCR2 antagonist and could block the CCL2‐CCR2 axis. 747 showed anti‐tumour activity by inhibiting TAM recruitment and elevating CD8^+^ T‐cell numbers. In addition, blockade of TAMs enhanced the anti‐tumour efficacy of sorafenib. The discovery of 747 provided a novel perspective on the development of CCR2 antagonists and enriched the therapeutic options against cancer.

##### Others

CCX872, a small molecule antagonist of CCR2, can impede MDSC invasion into tumour sites.^119^ The CCR2 inhibitor, PF‐04136309 can decrease tumour‐infiltrating inflammatory monocytes and macrophages, promoting anti‐tumour immunity in mice.^48^ Targeting CCR2 with CCX872 and PF‐04136309 for treating cancer has been investigated in clinical trials, which is shown in Table 2.

**TABLE 2:** Overview of clinical trials targeting the CCL2‐CCR2 axis

Target	Drug name	Conditions	Phase	Status	Trial number
CCL2	Carlumab	Patients with metastatic castrate‐resistant prostate cancer.	Phase II	Completed	NCT00992186
		Combination with chemotherapy in patients with solid tumours.	Phase I	Completed	NCT01204996
		Patients with solid tumours.	Phase I	Completed	NCT00537368
CCR2	MLN1202	Cancer patients with bone metastases.	Phase Ⅱ	Completed	NCT01015560
CCR2/CCR5	BMS‐813160	With or without GVAX for locally advanced pancreatic ductal adenocarcinomas.	Phase Ⅰ/Ⅱ	Recruiting	NCT03767582
		Combination with Nivolumab, Gemcitabine and Nab‐paclitaxel in borderline resectable and locally advanced pancreatic ductal adenocarcinoma (PDAC).	Phase Ⅰ/Ⅱ	Recruiting	NCT03496662
		Combination with chemotherapy or Nivolumab in patients with pancreatic cancer.	Phase Ⅰ/Ⅱ	Not recruiting	NCT03184870
		Combination treatments in patients with advanced renal cell carcinoma.	Phase Ⅱ	Recruiting	NCT02996110
		Combination treatments in patients with hepatocellular carcinoma.	Phase Ⅱ	Recruiting	NCT04123379
CCR2	CCX872‐B	Patients with pancreatic adenocarcinoma.	Phase Ⅰ	Not recruiting	NCT02345408
		Preoperative stereotactic body radiation therapy for pancreatic adenocarcinoma.	Phase Ⅰ/Ⅱ	Withdrawn	NCT03778879
CCR2	PF‐04136309	Patients with borderline resectable and locally advanced pancreatic adenocarcinoma.	Phase Ⅰ	Completed	NCT01413022
		Combination with Gem/Nab‐P in first‐line metastatic pancreatic patients.	Phase Ⅱ	Terminated	NCT02732938

#### 5.1.3 Combined therapy

The pathogenesis is complicated in cancer. Blockade of the CCL2‐CCR2 axis alone may cause unexpected side effects. Therefore, combining inhibition therapy of CCL2‐CCR2 axis with current treatment tactics for different cancers is necessary. In pancreatic adenocarcinoma, TANs and TAMs are recruited to the TME, mediating immune escape and favouring tumour growth. In patients with pancreatic adenocarcinoma, blockade of CCR2 prevented the accumulation of TAM in tumour sites but led to a compensatory CXCR2^+^ TANs influx, which possibly limited the intended treatment efficacy.^157^ A preclinical study reported a dual targeted strategy, where CCR2 inhibition combined with CXCR2 blockade resulted in notable tumour suppression and improved survival in tumour‐bearing mice. Further, dual targeting of CCR2^+^ TAMs and CXCR2^+^ TANs restored anti‐tumour immunity and improved the chemotherapeutic effect, providing the theoretical basis for targeting both CCR2 and CXCR2 in future clinical trials.^157^

The immune system is involved in almost every stage of cancer initiation and progression. Programmed cell death protein 1 (PD‐1) is an immune inhibitory receptor and is particularly expressed on cytotoxic T cells. PD‐1 plays a vital role in promoting self‐tolerance and inhibiting immune responses by binding to its ligand PD‐L1 or PD‐L2.^158^, ^159^, ^160^ Interestingly, recent studies have found that the CCL2‐CCR2 axis could induce immune escape through PD‐1 signalling in cancer.^102^ More recently, Fein and colleagues found that CCR2 secreted by tumour cells orchestrated immune evasion by depressing CD103^+^ DC maturation and suppressing CTL activity. Besides, CCR2^−/−^ cancer cells had lower surface levels of PD‐L1 compared with CCR2^+/+^ cancer cells.^161^ Combination therapy of CCR2 antagonist and anti‐PD‐1 also improved anti‐tumour effects compared with monotherapy.^162^, ^163^ It would be valuable to further explore the prospects of combining CCL2‐CCR2 axis blockade with PD‐1‐directed immunotherapies in multiple tumours.

### 5.2 Clinical trials targeting the CCL2‐CCR2 axis

The efficacy of CCL2‐CCR2 axis inhibition has been widely investigated in numerous preclinical experiments. CCL2 and CCR2 can function as diagnostic biomarkers,^164^ and are correlated with the prognosis and poor overall survival in cancer patients.^105^, ^165^ Given the critical roles of the CCL2‐CCR2 signalling axis in tumorigenesis, a series of clinical trials targeting this axis have been launched continually (Table 2).

#### 5.2.1 CCL2 neutralizing antibody

Targeting CCL2 is based on the assumption that CCL2 neutralizing antibodies can bind to CCL2 and block the recruitment and differentiation of macrophages. Carlumab (CNTO 888), a human anti‐CCL2 neutralizing antibody, can bind to human CCL2 and exhibits broad spectrum of preclinical anticancer activity.^166^ In a phase I study of the safety of carlumab in patients with solid tumours (NCT00537368), forty‐four patients with advanced solid tumours were enrolled. No dose‐limited toxicity (DLT) was observed in the carlumab‐treated patients, indicating that carlumab was generally well tolerated. However, in thirty‐three evaluable patients, none of them achieved an objective response, but durable stable disease (SD) was observed in four patients.^167^ In a phase II study of carlumab in metastatic castration‐resistant prostate cancer (NCT00992186), only 34% of patients maintained stable disease for more than three months, and none of the forty‐four patients evaluable for prostate specific antigen (PSA) response had a response. Notably, the concentrations of median free CCL2 sharply declined from baseline at 4 hours after carlumab treatment, but total CCL2 concentrations increased to 1000‐fold higher than baseline after one week.^168^ Similarly, in an open‐label, multicentre phase Ib study (NCT01204996), researchers found that carlumab sequestered CCL2 for only a short time and did not generate obvious anti‐tumour responses.^169^ The reasons for these unexpected results may be the weak affinity of carlumab with CCL2 and the inadequate clearance of the circulating CCL2‐complex.

Collectively, although no significant adverse reaction was found in these patients, the therapeutic effects of carlumab were not satisfactory. In the future, how to permanently suppress CCL2 levels may be the focus of research.

#### 5.2.2 CCR2 antibodies and inhibitors

Antibodies and inhibitors are used to interfere with CCR2 binding to its cognate ligand CCL2 and to block the activation of latent CCR2. Both steps are critical for CCR2‐induced pro‐tumorigenic effects and immune escape. CCR2‐associated targeted therapy has made progress in recent years.

##### PF‐04136309

PF‐04136309 is a CCR2 inhibitor that exhibited anti‐tumour activity in an orthotopic model of murine pancreatic cancer.^48^ Based on its anticancer potential, PF‐04136309 has been tested in clinical trials. A non‐randomized open‐label phase IIb study (NCT01413022) assessed the effect of PF‐04136309 in combination with FOLFIRINOX chemotherapy (oxaliplatin and irinotecan plus leucovorin and fluorouracil) in patients with pancreatic cancer. In all patients treated with FOLFIRINOX plus PF‐04136309, 32 patients (97%) achieved the control of local tumours, and 16 patients (49%) achieved an objective tumour response. Nevertheless, none of all patients achieved an objective response in the FOLFIRINOX chemotherapy alone group.^170^ What's more, after the treatment of FOLFIRINOX plus PF‐04136309, the mean ratio of blood to bone‐marrow CCR2^+^ monocytes in patients was significantly decreased compared with that in the FOLFIRINOX alone group (1.06 vs. 6.46), which indicated that FOLFIRINOX plus PF‐04136309 could prevent CCR2^+^ monocyte egress from the bone marrow to the peripheral blood and might affect the anti‐tumour immunity.^170^

Another phase I study (NCT02732938) was initiated to evaluate the effect of administering PF‐04136309 combined with gemcitabine and nab‐paclitaxel in patients with metastatic pancreatic cancer. All patients (21) received PF04136309 at a starting dose of 500 mg or 750 mg twice daily (BID). The final result showed that 14 patients (66.7%) experienced treatment‐related serious adverse events (SAEs), which might attribute to at least one of the three exposure drugs (PF‐04136309, nab‐paclitaxel, or gemcitabine). By the cut‐off date, the objective response rate (ORR) was 23.8% (95% CI = 8.2%‐47.2%) in all 21 patients who received the treatment, but the overall survival (OS) was not assessed in this trial. In the 500 mg BID group (n = 17), the median progression‐free survival (mPFS) was 5.3 months and the ORR was 29.4% (95% CI = 10.3%‐56.0%).^171^ Overall, PF‐04136309 in combination with chemotherapy showed clinically meaningful efficacy and survival benefit in patients with pancreatic cancer,^170^ but the value of PF‐04136309 in treating cancer patients remains to be further explored and more evidence for larger samples is also needed.

##### MLN1202

MLN1202 is an anti‐CCR2 monoclonal antibody. In a phase II clinical trial, researchers assessed the efficacy of MLN1202 in forty‐four patients with bone metastases (NCT01015560). Among forty‐three eligible patients, forty‐one patients completed the study, and only 7.14% had severe adverse reactions. To some extent, MLN1202 was well tolerated in patients. After forty‐three days of MLN1202 treatment, the urinary N‐telopeptide value (a primary assessment indicator) was decreased in 14 patients, indicating some positive effects of MLN1202 for cancer treatment.

##### Propagermanium

Propagermanium (3‐oxygermylpropionic acid polymer) is an organogermanium compound that blocks CCR2 signalling.^35^ In a phase I dose‐escalation study, a total of twelve patients with breast cancer were enrolled. The patients were all at tumour‐node‐metastasis (TNM) stage I, II, or III. After treatment with propagermanium, the serum IL‐6, which participated in tumour angiogenesis, was downregulated in a dose‐dependent manner, suggesting that propagermanium may have the potential to inhibit angiogenesis and prevent cancer metastasis. In the future, a phase II clinical trial of propagermanium will be conducted to assess its antimetastatic potential.^172^

##### CCX872‐B

CCX872‐B is a specific CCR2 antagonist. A phase Ib trial was designed to investigate the safety and efficacy of CCX872‐B in patients with pancreatic adenocarcinoma (NCT02345408). The enrolled patients (50) received FOLFIRINOX (fluorouracil [5‐FU], leucovorin, irinotecan, oxaliplatin) plus 150 mg CCX872 QD or BID for 12 weeks. From the perspective of survival improvement, all‐subjects overall survival (OS) at 18 months was 29%,^173^ whereas the OS was only 18.6% at 18 months for FOLFIRINOX regimen alone,^174^ suggesting that CCX872 might be a promising option for cancer treatment.

##### BMS‐813160

BMS‐813160 is a dual CCR2/CCR5 antagonist. Several clinical trials are currently evaluating the combination therapy of BMS‐813160 and chemotherapies or immunotherapies for treating cancer. (NCT 03767582, NCT03496662, NCT03184870, NCT02996110, NCT04123379) The anti‐tumour effects of BMS‐813160 have not been disclosed.

## 6 CONCLUSION

Although CCL2 was initially identified as a mediator of inflammation, increasing evidence has linked CCL2 with human diseases, particularly cancer. Deeper insight into the mechanisms of the CCL2‐CCR2 axis would provide new directions for better understanding of malignant tumours. The CCL2‐CCR2 signalling axis sustains primary tumour cell survival and proliferation through angiogenesis and different signalling pathways. Moreover, the axis can induce immunosuppressive cell entry to the TME, accelerating tumour growth and metastasis. The expression levels of CCL2 and CCR2 can be regarded as a predictor of poor prognosis in patients with cancer. Nevertheless, researchers noticed that high CCL2 expression was associated with favourable overall survival and progression‐free survival in patients with lung squamous cell carcinoma (LUSC). They speculated that the possible reason is that most TAMs differentiated into the M1 subtype in LUSC.^175^ Similarly, CCL2 secreted by hepatic macrophage can stimulate the establishment of an M1‐dominant hepatic macrophage phenotype.^176^

CCL2 neutralizing antibodies or CCR2 antagonists have been developed to potentiate anti‐tumour effects and have produced encouraging results in some preclinical studies. However, targeting CCL2 with carlumab did not appear to have a significant effect in clinical trials, suggesting that anti‐CCR2 may be a compensatory approach for the deficiency of targeting CCL2. In pancreatic cancer, CCR2 antagonists alone or combined with chemotherapeutic agents can control local tumours and are well tolerated in patients. Additionally, in several cancer models, scientists discovered that the CCL2‐CCR2 signalling axis could induce tumour immune evasion through PD‐1 signalling. Furthermore, the combined treatment of anti‐PD‐1 and inhibiting the CCL2‐CCR2 axis is more effective than monotherapy. It might be promising to move this combination therapy regimen into a clinical trial in the near future.

In conclusion, the CCL2‐CCR2 signalling axis covers a wide range of tumour cells' activities and is involved in both the early and late steps of tumorigenesis. A series of therapeutic strategies targeting this axis have enriched our approaches for fighting cancer and have entered clinical trials. However, it is important to note that discontinuing CCL2 inhibition exacerbates tumour metastasis,^177^ suggesting the need to develop new therapies to achieve long‐lasting CCL2 inhibition. Moreover, combination therapy of anti‐PD‐1 and CCL2‐CCR2 axis inhibition may be a novel and promising therapeutic approach for improving anti‐tumour efficacy, which provides new directions for cancer immunotherapy and warrants further study in clinical trials.

## CONFLICT OF INTEREST

The authors declare no competing financial interests.

## AUTHOR CONTRIBUTIONS

YQW and XWW offered main direction and significant guidance of this manuscript. MSX and YW drafted the manuscript. RLX illustrated the figures and tables for the manuscript. All authors approved the final manuscript.

## Data Availability

The data that support the findings of this study are available from the corresponding author upon reasonable request.
